# Ecological vulnerability indicators to drought: Case of communal farmers in Eastern Cape, South Africa

**DOI:** 10.4102/jamba.v11i1.591

**Published:** 2019-01-15

**Authors:** Andries Jordaan, Yonas T. Bahta, Boitumelo Phatudi-Mphahlele

**Affiliations:** 1 Disaster Management Training and Education Centre for Africa, University of the Free State, South Africa; 2 Department of Agricultural Economics, University of the Free State, South Africa

## Abstract

Estimation of ecological drought vulnerability indicators is the important step for drought mitigation management. This article identified and estimated ecological drought vulnerability indicators among communal farmers in the Eastern Cape province of South Africa, using an ecological vulnerability index based on a household survey of 121 communal farmers. The results identified overgrazing, soil erosion, land degradation, surface and groundwater supply, and land use management as the main ecological vulnerability variables. The results showed that climate is not necessarily linked to ecological vulnerability. High rainfall districts in this study showed higher ecological vulnerability to drought because of poor planning and management of water supply, poor grazing practices and land management that leads to serious land degradation. The identification and analysis of ecological vulnerability indicators to drought would aid in reconsidering priorities for the government to implement appropriate policy measures in response to drought and suggest strategies to reduce drought vulnerability. Such policies and strategies will strengthen climate change adaptation and ensure ecological and climate sustainability that comply with the Millennium Development Goals set out by the United Nations in 2000 and the subsequent 2030 development agenda for the Sustainable Development Goals.

## Introduction

Drought is a normal recurring event that affects people around the world and is one of the most important natural disasters in economic, social and ecological terms (Buckland, Elele & Mugwara 2000; Ranger, Harvey & Gabrett-Shiels 2014). The Eastern Cape (EC) province is highly vulnerable to disaster because of a high level of poverty, low standards of living, environmental degradation, poor household economies and a lack of access to resources (Bahta, Jordaan & Muyambo 2016). The EC not only has the biggest cattle and sheep herds in South Africa but also has the practice of communal farming on the largest scale in the country (Nowers 2008). Everybody in agriculture acknowledges climatic extremes and the fact that they will experience future dry and wet periods. It is just a matter of when and how severe (Jordaan, Sakulski & Jordaan 2013). The uncertain and erratic nature of wet periods is related to uneven distribution of rainfall. Prolonged dry periods lead to complete losses of yield, herds and capital; to psychological stress; and even to a loss of farmers’ lives (Bahta et al. 2016; Edwards, Gray & Hunter 2015; Obrien et al. 2014). These uncertainties greatly affect communal and small-scale farmers. Communal and small-scale farmers in South Africa are particularly vulnerable to drought shocks, and they experience normal dry periods as drought disasters (Jordaan 2011; Jordaan et al. 2013). The impact of drought results in a recurring deficiency of food supplies, and the need arises for interventions by government and international donors to alleviate food shortages to prevent loss of human life (Botterill & Fisher 2003).

According to Global Crisis Solution (2014), vulnerability is explained as a set of significant conditions, which badly affects the community’s ability to prevent, mitigate and prepare for a response to hazardous events. A vulnerability can be measured by considering social, economic and ecological factors, and this article will only focus on the ecological vulnerability to drought. The ecological vulnerability is a key factor that defines the impact of drought; the more vulnerable the ecology, the greater the potential losses (Blaikie et al. 1994). Identifying ecological vulnerability to drought is a crucial step in managing the risk of drought and can support mitigation-oriented management (Wilhelmi & Wilhite 2002). The Hyogo Framework for Action (2005–2015) highlights the importance of social, economic and environmental or ecological^1^ vulnerabilities to disasters and promotes policy, planning and action with a focus on these spheres of disaster hazard impact. The importance of vulnerability indicators is also emphasised by the Hyogo Framework as a ‘key activity’ by stating (United Nations 2005):

the need for the development [of a] system of indicators of disaster risk and vulnerability at national and sub-national scales that will enable decision makers to assess the impact of the disaster on social, economic and ecological conditions and circulate the results to decision makers, the public and population at risk. (p. 7)

Various methods have been employed to estimate ecological vulnerability indicators. Li et al. (2009) applied an eco-environmental vulnerability assessment using integrated fuzzy analytic hierarchy process (FAHP) and geographic information system (GIS) that was developed for the Danjiangkou reservoir area. Similar international studies explain the use of vulnerability indices. Examples are coastal city flood vulnerability index, flood disaster using data envelope analysis method, hydrological vulnerability index, social vulnerability to climate change and socio-environment system environmental variability, vulnerability of people, places to environmental and social force, the complex relationship between environmental risk, poverty and vulnerability, composite vulnerability indicator and vulnerability of socio-environmental system (Balica, Wright & van der Meulen 2012; Brouwer et al. 2007; Cutter 1996; Eakin & Lures 2006; Jun et al. 2011; Li et al. 2009, 2013; Luers 2005; Mdungela, Bahta & Jordaan 2017; Meier, Bond & Bond 2007; Muyambo, Jordaan & Bahta 2017; Naumann et al. 2014; Vincent 2004). Few studies in South Africa explain the use of indicators for vulnerability assessment. Therefore, this study attempts to fill this gap in knowledge and literature.

The research which is reported in this article is part of a more comprehensive research project titled ‘Vulnerability, adaptation and coping with drought: The case of the commercial and subsistence extensive livestock sector in the Eastern Cape’ (Jordaan et al. 2017). This report focuses only on the ecological vulnerability indicators to drought among communal farmers. Drought will continue to pose a threat to farmers and the community at large, and thus, the effects should be mitigated and monitored to lessen the impacts. This study adds a scientific contribution, which aid decision- and policy-makers to formulate appropriate policy interventions to sustain communal farmers against the perils of drought, which is a threat to food security, human survival and living standards of farmers. Finally, the recommendations, if implemented, will ensure a sustainable ecological environment required for a resilient communal farming sector. Although this study was applied in South Africa, the framework has the potential to be replicated in different case studies.

## Study area

**FIGURE 1:**
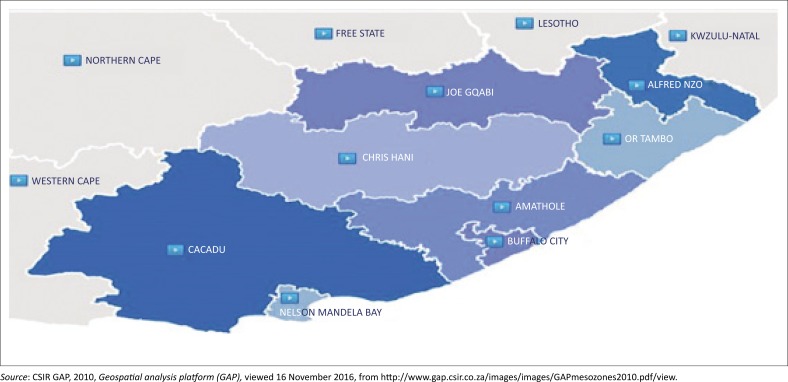
Eastern Cape province.

The Eastern Cape is the second largest province, following the Northern Cape in South Africa, and it covers close to 169 000 km^2^ (Hamann & Tuinder 2012). The province makes up 13.5% of South Africa’s total population (Statistics South Africa 2012). The research was conducted in three of the district municipalities, namely Cacadu, Joe Gqabi and OR Tambo district municipalities. A map of the Eastern Cape province with the various regions is shown in Figure 1.

The mentioned districts were selected mainly because of the large variation in climate. The eastern parts of OR Tambo receive more than 1000 mm precipitation per annum with less than 300 mm in the western parts of Cacadu district. Drought is also a recurring drought event in all three districts. Additionally, these districts were also selected because communal farming is practiced in large scale and is still managed by chiefs or communal leaders.

## Research methodology

Primary data were collected using a semi-structured questionnaire from 121 communal farmers who were interviewed face to face. The study adopted a multistage sampling approach. The first stage involved the purposive sampling of three districts from the Eastern Cape province. The second stage involved the proportional sampling of communal farmers (Kothari 2004). We proportionally sampled 19 communal farmers from Joe Gqabi, 15 communal farmers from Cacadu and 87 communal farmers from OR Tambo district municipalities. The difference in sample sizes selected from the different districts was based on the proportion of communal farmers in the district municipalities. We sampled more farmers from OR Tambo district because of its large number of communal farmers, relative to Cacadu and Joe Gqabi districts. In total, 121 farmers were sampled and interviewed during the period from August to September 2014. As part of getting an understanding of the vulnerability situation in the study area, a qualitative Rapid Rural Appraisal (RRA) approach was also applied together with a transect drive through all three districts.

### Data analysis

The survey data collected from the sampled respondents were processed and used in estimating the ecological vulnerability index. Prior to the estimation of the vulnerability index, summary statistics of the communal farmers were described to give an overview of the socio-economic characteristics of respondents. Among the socio-economic characteristics are age, gender, educational background, household size, access to resources and farming experience. The collected data were analysed using descriptive statistics of percentage and frequency. These descriptive statistics were performed on data such as age, gender, educational background, household size, access to resources and farming experience.

**FIGURE 2:**
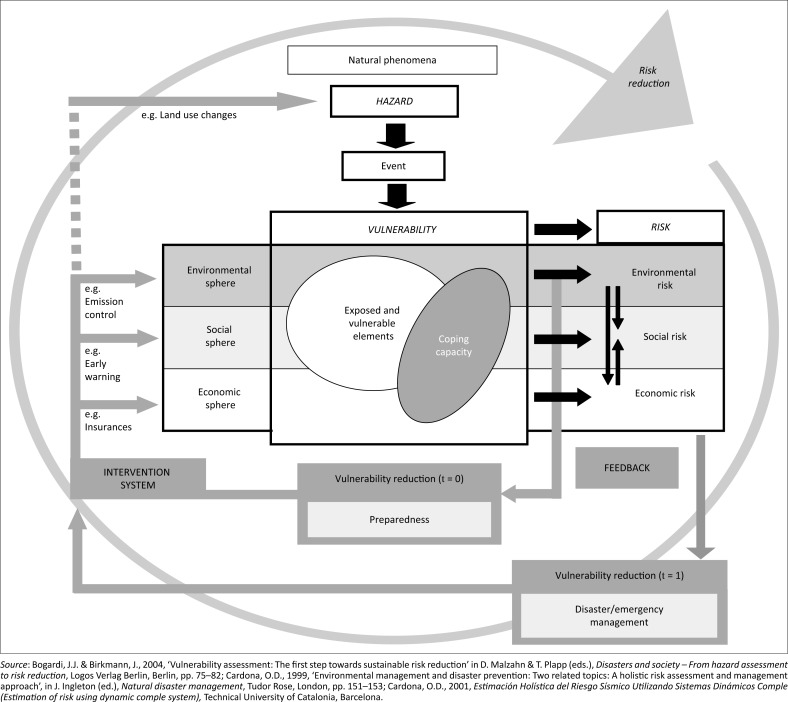
Conceptual framework for vulnerability.

We first identified each municipality’s ecological vulnerability indicators for drought using the BBC (Bogardi, Birkman, Cardona) framework (Figure 2). The term ‘BBC’ framework was derived from their work of conceptual models (Bogardi & Birkmann 2004; Cardona 1999, 2001). The BBC conceptual framework highlights the complexity of vulnerability and resilience to external shocks, humans and the ecological. The BBC framework was selected because it addresses various vulnerabilities in the social, ecological and economic spheres. The three spheres are important pillars of sustainable development. The BBC model was also preferred in this study for its promotion of proactive action in risk reduction. It demonstrates the necessity of having intervention strategies in place prior to the occurrence of a disaster (Bogardi & Birkmann 2004; Cardona 1999, 2001).

This study focuses on the ecological or environmental aspect only. The selection of ecological indicators was complex because of the multifunctional nature of ecological indicators (Donnelly et al. 2007; Kurtz, Jackson & Fisher 2001).

Empirically, the ecological vulnerability index was specified as:

$$V^{eco}=\sum_{i=1}^{8}w_{i}^{eco}v_{i}^{eco}.$$ [Eqn 1]

$$V^{eco}f(w_{1}^{eco}v_{1}^{eco},w_{2}^{eco}v_{2}^{eco},w_{3}^{eco}v_{3}^{eco},...........w_{5}^{eco}v_{5}^{eco})$$ [Eqn 2]

where

$v_{1}^{eco}$ = overgrazing

$v_{2}^{eco}$ = soil erosion

$v_{3}^{eco}$ = land degradation

$v_{4}^{eco}$ = surface and ground water supply

$v_{5}^{eco}$ = land use management practice

$w_{1}^{eco}....w_{5}^{eco}$ = equal weighting factor for all variables or indicators.

Table 1 presents the ecological indicators that were selected. The factors considered in the selection of ecological indicators for drought were (1) expert opinion, (2) observations, (3) ease of measurement, (4) feedback from farmers, (5) relevance, (6) availability of data and (7) the importance of the indicator for vulnerability or resilience measurement. Vulnerability indicators were measured on a Likert-type scale where vulnerability index was rated as follows:

- 1 = resilient
- 2 = slightly vulnerable or resilience
- 3 = moderately vulnerable
- 4 = highly vulnerable
- 5 = extremely vulnerable.

**TABLE 1:** Classification criteria of selected ecological vulnerability indicators.

Ecological indicators	Index (Likert scale)	Description of indicator classification	Statement of measurement	Relationship with vulnerability	Data source
Overgrazing	1	Zero land overgrazing	Percentage of affected grass cover	As grazing pressure increase the land is more vulnerable	Survey and Observation
	2	Moderate overgrazing in some areas			
	3	Serious overgrazing in some areas			
	4	Serious overgrazing in large areas			
	5	Total area seriously overgrazed			
Soil erosion	1	100% excellent, no soil erosion	Percentage of soil eroded in a period of 30 years	The greater the extent of soil erosion the greater the vulnerability	Survey and Observation
	2	Few examples of erosion detected			
	3	Moderate erosion in some areas			
	4	Serious erosion in some areas			
	5	Serious erosion in most areas			
Land degradation	1	Slightly degraded	Proportion of degraded area a period of 30 years	The more degraded the land the more vulnerable	Survey and Observation
	2	Moderate			
	3	High			
	4	Very high			
	5	Severe			
Land use and land management practices	1	Very well planned in total area	Extent of land use planning	The less well planned the land is- the greater the vulnerability	Survey and Observation
	2	Well planned in most of the area			
	3	Planned but large areas not planned			
	4	Poorly planned in most of the area			
	5	No planning at all			
Surface and groundwater supply	1	Groundwater and surface water always available everywhere	The amount of available water in the recharged area	The higher the groundwater supply the greater the coping capacity	Observation
	2	Both groundwater and surface water available at most places during drought			
	3	Either groundwater or surface water available at some places during drought			
	4	Limited amounts of groundwater or surface water available at some places during droughts			
	5	No groundwater or surface water supply during drought			

*Source:* Jordaan, A.J., Muyambu, F., Mdungela, N., Phatudi-Mphahlele, B., Bahta, Y.T., Mashimbye, C., et al., 2017, ‘Drought vulnerability: Communal farmers’, in A.J. Jordaan (ed.), *Vulnerability, adaptation to and coping with drought: The case of commercial and subsistence rain fed farming in the Eastern Cape, vol. II,* pp. 6.47– 6.55, WRC Report No. TT 716/2/17, ISBN 978-1-4312-0885-2, Water Research Commission (WRC), Pretoria.

## Result and discussion

### Socio-economic aspects of the respondents

One hundred and twenty-one communal farmers were interviewed from Joe Gqabi (*n* = 19), Cacadu (*n* = 15) and OR Tambo (*n* = 87) district municipalities. The demographic and socio-economic characteristics are important because they influence households’ economic behaviour (Randela 2005). Some of the socio-economic characteristics of the respondents are provided in Table 2. Most respondents were male (73%). A possible reason for the male-dominated farming activities in the study area might be that they had access to land. Quisumbing (1994) reported that there is a great disparity between women and men in the size of landholdings and that the mode of women participation in agricultural production varies with the land-owning status of households.

**TABLE 2:** Socio-economic characteristics of the respondents.

Characteristics	Sub-characteristics	OR Tambo (*n* = 87)		Joe Gqabi (*n* = 19)		Cacadu (*n* = 15)		% (*N* = 121)			Total (%)
		*n*	%	*n*	%	*n*	%	ORT	JG	CD	
Age (years)	25–34	7	8	3	16	2	13	6	2	2	10
	35–44	20	23	3	16	2	13	17	2	2	21
	45–54	25	29	4	21	4	27	21	3	3	27
	> 55	35	40	9	47	7	47	29	7	6	42
	Sub-total of age	87	100	19	100	15	100	73	14	13	100
Gender	Male	62	71	16	84	11	73	51	13	9	73
	Female	25	29	3	16	4	27	21	3	3	27
	Sub-total of gender	87	100	19	100	15	100	72	16	12	100
Education	None	23	26	1	5	3	20	19	1	3	23
	Primary	44	51	13	68	12	80	36	10	10	56
	Secondary	18	21	2	11	-	-	15	2	-	17
	Graduate	2	2	3	16	-	-	2	2	-	4
	Sub-total of education	87	100	19	100	15	100	72	15	13	100
Household size	0–4	29	33	5	26	8	54	24	4	7	35
	5–8	32	37	11	58	5	33	26	9	4	39
	9–12	14	16	3	16	2	13	12	2	2	16
	> 13	12	14	-	-	-	-	10	-	-	10
	Sub-total of household	87	100	19	100	15	100	72	15	18	100
Access to resources	Land	69	79	16	84	9	60	57	13	7	77
	Not access	18	21	3	16	6	40	15	3	5	23
	Sub-total of land	87	100	19	100	15	100	72	16	12	100
Access to resources	Water	33	38	10	53	6	40	28	8	5	41
	Not access	54	62	9	47	9	60	45	7	7	59
	Sub-total of water	87	100	19	100	15	100	73	15	12	100
Experience (years)	0–4	10	12	4	21	7	47	8	3	6	17
	5–9	20	23	6	32	4	27	17	5	3	25
	10–14	28	32	4	21	2	13	23	3	2	28
	> 15	29	33	5	26	2	13	24	4	2	30
	Sub-total of experience	87	100	19	100	15	100	72	15	13	100

*Source*: Jordaan, A.J., Muyambu, F., Mdungela, N., Phatudi-Mphahlele, B., Bahta, Y.T., Mashimbye, C., et al., 2017, ‘Drought vulnerability: Communal farmers’, in A.J. Jordaan (ed.), *Vulnerability, adaptation to and coping with drought: The case of commercial and subsistence rain fed farming in the Eastern Cape, vol. II,* pp. 6.47–6.55, WRC Report No. TT 716/2/17, ISBN 978-1-4312-0885-2, Water Research Commission (WRC), Pretoria.

ORT, OR Tambo; JG, Joe Gqabi; CD, Cacadu district municipality; *n*, number.

Many respondents (23%) did not have a formal education, 17% had a secondary level education and only 4% had a tertiary education. Education level is of importance, as this can influence households’ behaviour (Randela 2005). The results showed that communal farmers in OR Tambo (79%), Joe Gqabi (84%) and Cacadu district (60%) had access to land. In general, 77% of the respondents had access to land, of whom 57% were from OR Tambo, 13% Joe Gqabi and 7% from Cacadu district municipalities. Thirty-eight per cent of communal farmers from OR Tambo, 53% from Joe Gqabi and 40% from Cacadu district municipalities had access to water. Forty-one per cent of respondents had access to water, the majority (28%) from OR Tambo district municipality, 8% from Joe Gqabi and 5% from Cacadu district. Of the respondents, 42% were 55 years or older, 39% had a household size of 5–8 inhabitants and 58% had more than ten years of farming experience.

The land was owned by the community and managed by an elected committee or was held by a community leader or governmental authority. The practice system renders farmers vulnerable in the sense that financial institutions are reluctant to lend money without individual title deeds to be used as collateral security. Respondents confirmed that the lack of land ownership was a huge problem and increased their vulnerability to drought. Farmers also had to keep animals on small pieces of land, resulting in overgrazing, soil erosion and land degradation.

The unavailability of farming equipment increases farmers’ vulnerability to drought. Respondents acknowledged that they did not have equipment for their farming enterprises because such equipment is expensive and out of reach for communal farmers. A possible solution is that government could assist communal farmers in accessing equipment in order to reduce their vulnerability to drought. Farm inputs, such as fertilisers, pesticides, grazing land, and improved seeds or cultivars, are also important in increasing the resilience of the farmers.

### Ecological vulnerability analysis

Zuma-Netshiukhwi, Stigter and Walker (2013) mentioned that farmers regularly experience destructive disasters that are weather and climate related, for example, floods, below average rainfall, severe dry periods, and strong winds that contributed and intensified veld fire impacts, while Knutson, Hayes and Phillips (1998) stated that the lack of water during drought increased the difficulty of fighting veld fires.

It was recognised that farmers were aware and concerned about ecological changes and damages that affected agricultural production such as soil erosion, overgrazing and land degradation. They understood that the physical environment is deteriorating. Ecological indicators that were identified in the study area were (1) overgrazing, (2) soil erosion, (3) land degradation, (4) surface and groundwater supply and (5) land use. Forty-four per cent of farmers in OR Tambo, Joe Gqabi and Cacadu districts reported insufficient water supply during dry periods.

#### Overgrazing

Overgrazing was one of the major ecological indicators for drought in the study area and is described as a shortage for pasture to livestock and a failure to match animal grazing to forage growth and production. Overgrazing arose as a result of having too many animals on the land or not properly controlling grazing activities. It reduced ground cover and also increased the likelihood of crusting conditions during rainy periods. The crusting conditions decreased water infiltration and prolonged plant recovery from previous droughts.

Overstocking, the absence of grazing management practices and lack of infrastructures such as fences and water reticulation systems were among the main reasons for severe land degradation in OR Tambo district and the eastern part of Joe Gqabi, despite relatively high rainfall. The Sterkspruit region in Joe Gqabi was also severely overgrazed with extreme erosion evident. Joe Gqabi and Cacadu were characterised by heavily overgrazed land on municipal land surrounding all the towns. The rest of Joe Gqabi and Cacadu were fairly well managed, with most commercial landowners mentioning an increase in vegetation cover since the livestock reduction schemes of 1982–1983 and 1992–1993.

**FIGURE 3:**
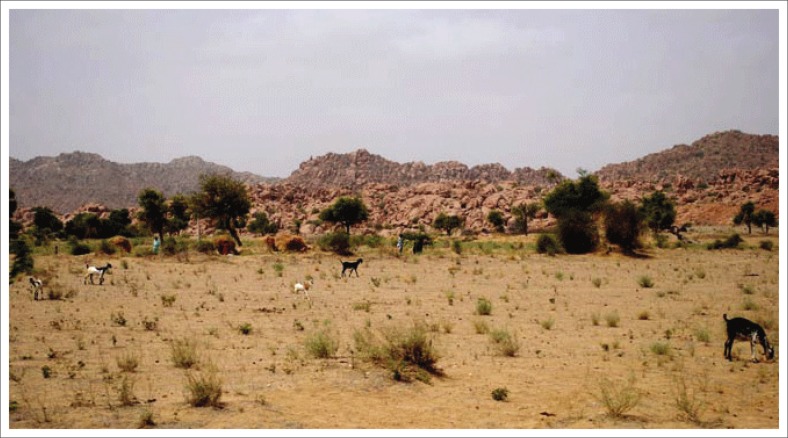
Overgrazed and degraded land.

The lack of grazing systems increased vulnerability to drought. In some cases, communal farmers did apply a rotational system of six months whereby they allowed certain areas to rest for 6 months. In Joe Gqabi and Cacadu districts, the findings show that rotational camps only rest for 6 months. Animals were rotated in camps or under the supervision of herders between summer and winter, but this was not sufficient to allow re-vegetation and proper re-growth. When rotational grazing camps are properly demarcated and planned, it allows the grass to recover (Snyman 2003). At the core of the problem was the lack of infrastructures such as grazing camps and water reticulation systems. Where infrastructure was in place, for example, at communal municipal land, the problem remained because land management plans were not enforced. Figure 3 shows affected overgrazed pastures.

In OR Tambo, it was observed that 20% of the land is overgrazed which was caused by overstocking of animals, while 16% in Joe Gqabi and 14% in Cacadu districts were because of poor livestock management. Figure 3 shows affected overgrazed pastures and this gave a clear motive that farmers need to allow sufficient recovery periods before the next grazing. Farmers in OR Tambo were receiving average precipitation despite that grazing lands are not doing well because they have not rested.

The effect of overgrazing in the OR Tambo district was dramatic. Virtually, no camps or proper water reticulation systems were available. Communal farmers, therefore, depended on the skills of herders to move animals between water points and towards areas where grazing was available. As a result, OR Tambo area was the most vulnerable to drought in spite of the fact that it is the district with the highest annual precipitation. The Sterkspruit area in Joe Gqabi was also classified as extremely overgrazed and vulnerable.

#### Soil erosion

Soil erosion was identified as one of the important ecological indicators for drought. Erosion is the detachment and transportation of soil materials by water and wind. As much as 70% of South Africa is affected by different types and levels of soil erosion (Le Roux et al. 2008; Garland, Hoffman & Todd 1999). Sheet, rill and gully erosion were the three most prominent types of erosion in the study area. Sheet erosion is the detachment of soil particles by raindrop impact and transportation by a shallow overland flow. Rill erosion describes the process where numerous small channels of up to 30 cm are formed (Lal & Elliot 1994). Gully erosion describes the process where surface water concentrates in narrow footpaths and transports the soil in channels that are too large to flatten with normal tillage operations (Kirby & Bracken 2009). These small gullies eventually develop into large gullies. The Eastern Cape is the province most severely affected by sheet and rill erosion with 6 188 581 hectares affected. It is also the province with most gully erosion at 151 759 ha affected, second only to the Northern Cape (160 885 ha). Le Roux (2011) named the most important factors influencing erosion as (1) climate erosivity, (2) soil erodibility, (3) slope gradient and length, (4) topography, (5) vegetation cover, (6) rainfall, (7) lithological factors, (8) pedological factors, (9) land use and (10) land management. Beyene (2011) urged that soil erosion is a global environmental problem that causes loss of fertile topsoil. Agricultural productivity is severely affected by eroded areas, and land is especially vulnerable to dry periods. Farmers farming on eroded soil are extremely vulnerable to droughts and even to normal dry periods.

**FIGURE 4:**
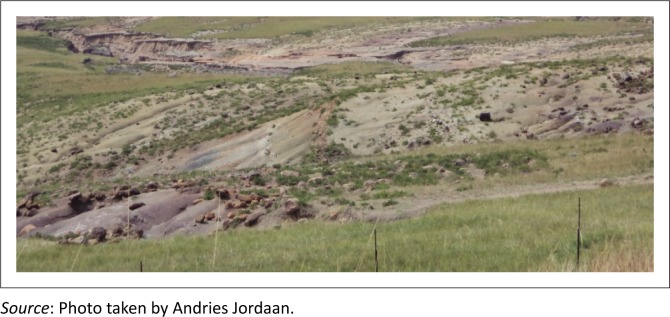
Soil erosion on sloped areas.

**FIGURE 5:**
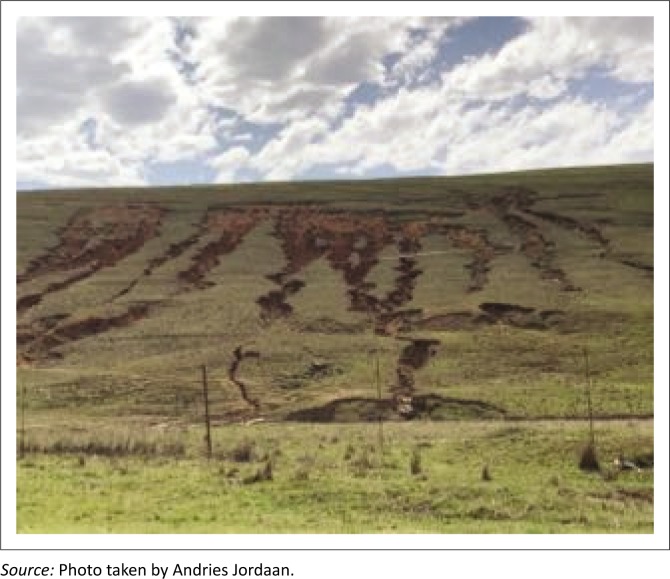
Soil erosion on sloped areas.

Soil erosion was evident in all three districts, but the Sterkspruit area and OR Tambo are possibly the most eroded areas in South Africa. In Tsolo and Umtata, there was 20% of soil erosion, and in Lusikisiki and Port St Johns, it was 13%. In Cacadu district, Graff-Rienet, Aberdeen and Willowmore were 15% eroded. In Joe Gqabi, Jamestown was observed to have 10%, while Barkly East, Ugie and Maclear were eroded by 12%. Figures 4 and 5 show an example of soil erosion in OR Tambo district. Agricultural production is adversely affected by eroded land. Through observations and feedback from farmers in all three districts, it became clear that farmers who farm in areas with high soil erosion were not able to cope with dry periods. Soil erosion is indicative of overgrazing and poor management practices and was used in the research as an indicator of drought vulnerability. The lack of vegetation growth is clearly illustrated in Figures 4 and 5.

#### Land degradation

**FIGURE 6:**
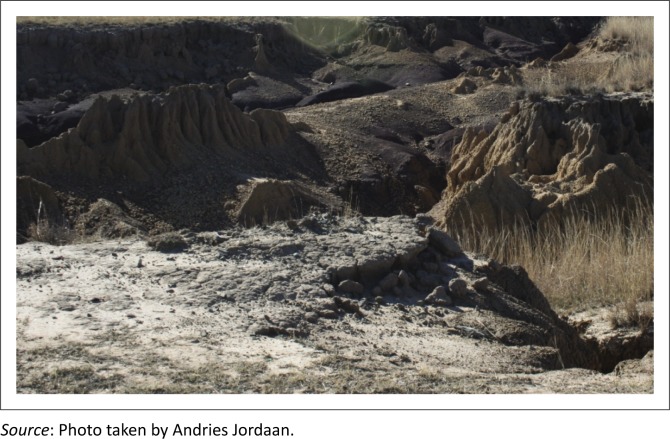
Severely degraded land in Mfolozi village near Tsolo.

Land degradation is normally characterised by soil erosion, lack of vegetation or invasive species (Snyman 2003). The land was degraded in all three districts, but more so in OR Tambo. Figure 6 shows an example of severely degraded land in Tsolo near Umtata. Wessels (2005) also concluded that the communal farming system was at the root of land degradation in OR Tambo and the rest of the Eastern Cape. The central and western regions of OR Tambo, namely the Tsolo and Umtata regions, were more degraded compared to the coastal areas at Lusikisiki and Port St Johns. Hoffman et al. (1999) supported this in their national review on land degradation in South Africa.

Degraded land in Cacadu and Joe Gqabi was more visible on municipal land around towns and again this was linked to the communal farming system. In Cacadu district, it was observed that 2% of land outside Willowmore was degraded, and Hoffmann et al. (1999) mentioned that there was insignificant land degradation in all of Cacadu except on communal land.

Land degradation as an ecological problem predisposes farmers to the adverse impacts of drought. In the badly degraded areas, agriculture was affected negatively as vegetation cannot grow, resulting in low potential grazing for animals. Farmers in such areas were more vulnerable to drought when compared to farmers in areas where there was no land degradation.

#### Surface and groundwater supply

**FIGURE 7:**
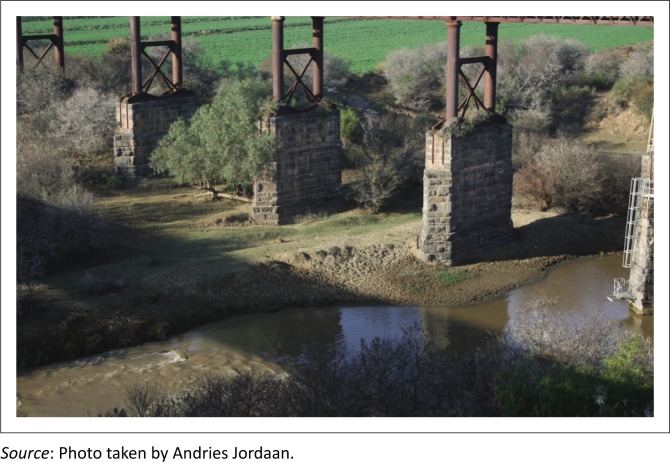
The Great Fish River on R67 towards Fort Beaufort in the Cacadu district.

The disappearance or drying up of surface and groundwater made farmers more vulnerable to drought. The level of groundwater supply (e.g. springs, boreholes and wells), surface water (e.g. rivers and streams) and dams in the study area were of great concern. The effects of severe dry spells on both the surface and groundwater became evident during the later stages of the research in 2016 when the Eastern Cape experienced a dry period. A large number of dried up wells and streams were reported while flow in rivers dropped dramatically and major dam levels were very low. For example, the Great Fish River (among others) in Cacadu district had almost dried up (Figure 7). OR Tambo district received average precipitation during the same period, but most of the water was not harvested and ended up in the ocean unused. Thirteen (13%) of the farmers in OR Tambo requested for dams to be built.

Surface water and groundwater supply get recharged when water from rainfall is absorbed into the ground. The failure to do so increases farmers’ vulnerability to drought. Peters et al. (2005) mention that the performance of groundwater systems under dry conditions is becoming imperative. No evidence was found of groundwater recharge in the study area in spite of a high dependence on groundwater in the western parts of the study area.

#### Land use and management practices

Examples of land use or land management practices that lead to land degradation and soil erosion were (1) removal of trees for agriculture, housing or other needs; (2) overgrazing because of too many animals or poor grazing practices; (3) cultivation on steep slopes; (4) disregard for water and soil conservation practices; (5) high per capita water consumption; and (vi) poor water run-off planning in developmental projects. These were identified as vulnerability indicators to drought in the study area. Food crops were grown on shallow and low potential soils, soils with stones or soil on steep slopes. The use of low potential soil for crop production or horticulture increases drought vulnerability. Grazing by livestock in rough pastures, mixed scrub or wooded areas alters and degrades vegetation zones, accelerates soil and nutrient loss and renders areas susceptible to the negative impacts of drought. The results of the research showed poor land use and land management practices in most of the communities in the study area. In OR Tambo district, there is no planning at all in land management practice, and in Joe Gqabi district, only few farmers applied land management practices; however in Cacadu district, well planned land management is practiced in most of the areas. Wilhite (2000) and Jordaan (2011) also reported poor land use management as a major contributor to land cover depletion and ultimately drought vulnerability.

### Estimation of ecological vulnerability indicators

Table 3 shows calculations for ecological vulnerability to drought. Each indicator was calculated using index values from 1 to 5 for selected indicators. Index values were allocated according to the *Classification criteria for selected vulnerability indicator* shown in Table 1.

**TABLE 3:** Estimation of ecological vulnerability indicators.

Indicators	OR Tambo district	Index	Joe Gqabi district	Index	Cacadu district	Index
Overgrazing	Most of the district overgrazed	5.0	Serious overgrazing in some areas (Sterkspruit, municipal land, Mount Fletcher)	4.0	Serious overgrazing in some areas (municipal land)	3.0
Soil erosion	Serious erosion in most areas	5.0	Serious erosion in some areas	5.0	Moderate erosion in some areas	3.0
Land degradation	Very high land degradation	5.0	Highly degraded	3.0	Moderately degraded	2.0
Surface and ground water	Either groundwater or surface water available at some places during drought	2.0	Either groundwater or surface water available at some places during drought	3.0	Limited amounts of groundwater or surface water available at some places during droughts	4.0
Land use management	No planning at all	5.0	Planned but large areas not planned (Sterkspruit, Mt Fletcher)	3.0	Well planned in most of the area	2.0

**Total score**		**22.0**		**18.0**		**14.0**
**EcoVI (total score ÷ no. of variables)**		**4.4**		**3.6**		**2.8**

*Source*: Jordaan, A.J., Muyambu, F., Mdungela, N., Phatudi-Mphahlele, B., Bahta, Y.T., Mashimbye, C., et al., 2017, ‘Drought vulnerability: Communal farmers’, in A.J. Jordaan (ed.), *Vulnerability, adaptation to and coping with drought: The case of commercial and subsistence rain fed farming in the Eastern Cape, vol. II,* pp. 6.47–6.55, WRC Report No. TT 716/2/17, ISBN 978-1-4312-0885-2, Water Research Commission (WRC), Pretoria.

Index, 1 = Resilient; 2 = slightly vulnerable or resilience; 3 = moderately vulnerable; 4 = highly vulnerable; and 5 = extremely vulnerable.

no., number.

The results highlighted the ecological vulnerability to drought in OR Tambo district in spite of the fact that it was the highest rainfall area. One would expect the more arid regions to be more vulnerable, which was not the case. Certain areas in other districts were also extremely vulnerable, such as all the municipal land and communal land in Joe Gqabi district. The commercial farming areas are reasonably resilient against drought because of proper vegetation cover and well-developed infrastructures such as fencing and water reticulation systems.

The mean vulnerability index value of 4.4 for OR Tambo is an indication of extreme ecological vulnerability for this district. Joe Gqabi district was also highly vulnerable to an index value of 3.6, and Cacadu was moderately vulnerable.

It is important, however, to note that the values indicated are for the districts as a whole but with a larger focus on communal land. The communal farmers who farm on communal land were all categorised as highly to extremely vulnerable. All communal farmers who participated in workshops and in questionnaires were of the opinion that they are extremely vulnerable to drought and that they needed government support in order to survive dry periods. Ecological vulnerability for commercial farms, on the other hand, was much lower because of better vegetation growth and less soil erosion and therefore ultimately more resilience against droughts.

## Conclusion and recommendations

Worldwide drought has a significant impact and continues to pose long-lasting effects on the agricultural sector. There are several factors that shape the onset of a drought and that has the capability to increase the effects of a drought. An ecological vulnerability is one such factor. One cannot influence rainfall patterns, but it is possible to prepare and build resilience against drought periods once the vulnerability factors are identified and known. The vulnerability to a drought of communal farmers was not linked to a single problem such as land management, but rather to a combination of many multi-disciplinary factors.

As land degradation and surface and groundwater were the key vulnerability indicators, we recommend that communal farmers should be trained and educated on how to conserve and utilise natural resources and community capitals. In order to sustain their livelihood, communal farmers should adapt a multi-disciplinary approach in the management of natural resources by incorporating natural capital (e.g. soil and water supply), human capital (e.g. enhancing communal farmer’s skill, health and disseminating information), social capital (e.g. networks), financial capital (e.g. access to funding), cultural capital and political capital (e.g. communal farmers access to public resources).

The extension services and, particularly, extension officers should play a major role in drought risk reduction through the application of well-designed extension programmes. The government should support the establishment of districts soil and environmental conservation committees coordinate, and monitor strategies to combat soil erosion, land degradation and overgrazing. Moreover, main key role players (Department of Agriculture, Forestry and Fisheries [DAFF] at the national level, provincial Departments of Agriculture, National and Provincial Disaster Management Centres [NDMC and PDMC], Department of Water Affairs [DWA], South African Weather Service [SAWS]) should work together and develop a sound natural resource management policy to reduce drought risk.

## Footnotes

1 Ecological is used in this article instead of environmental.
